# Entropy as an Objective Function of Optimization Multimodal Transportations

**DOI:** 10.3390/e23080946

**Published:** 2021-07-24

**Authors:** Oleg Bazaluk, Sergiy Kotenko, Vitalii Nitsenko

**Affiliations:** 1 Belt and Road Initiative Institute for Chinese-European Studies, Guangdong University of Petrochemical Technology, Maoming 525000, China; bazaluk@ukr.net; 2 Institute of Market Problems and Economic-Ecological Research, National Academy of Sciences of Ukraine, 65044 Odesa, Ukraine; svkotenko@yahoo.com; 3 Department of Economics and Finance, Educational and Scientific Institute of Marine Business, Odesa National Maritime University, 65029 Odesa, Ukraine; 4 SCIRE Foundation, 00867 Warsaw, Poland

**Keywords:** entropy method, multimodal transportations, mathematical model, transportation risks, various criteria optimization, fuzzy, determination and stochastic parameters

## Abstract

This article considers the use of the entropy method in the optimization and forecasting of multimodal transport under conditions of risks that can be determined simultaneously by deterministic, stochastic and fuzzy quantities. This will allow to change the route of transportation in real time in an optimal way with an unacceptable increase in the risk at one of its next stages and predict the redistribution of the load of transport nodes. The aim of this study is to develop a mathematical model for the optimal choice of an alternative route, the best for one or more objective functions in real time. In addition, it is proposed to use this mathematical model to estimate the dynamic change in turnover through intermediate transport nodes, forecasting their loading over time under different conditions that also include long-term risks which are significant in magnitude. To substantiate the feasibility of the proposed mathematical model, the analysis and forecast of cargo turnover through the seaports of Ukraine are presented, taking into account and analysing the existing risks.

## 1. Introduction

Multimodal cargo transportation involves the existence of a chain of successive transportations using different modes of transport. However, during transportation, it may turn out that at one of the stages of the route, the circumstances of transportation have become less favorable or extremely unfavorable, which is much worse, unacceptably increasing the risk of transportation. These risks are determined by many parameters that can be measured on different scales.

The task of finding a solution through many criteria and many parameters has found a wide response in the scientific literature. In particular, Ehrgott’s work is often referred to [1,2]. The classic article [1] rightly points out the complexity of optimization problems and proposes an application of the heuristic approach as a forced solution. The heuristic approach by definition does not give significant relevance to the proposed solutions. The article [2] proposes double multi-objective linear programming to simplify the calculation of optimization problems by their linearization. It should also be borne in mind that the approach [2] is proposed to avoid the use of classical optimization methods. This was taken into account when developing the mathematical approach proposed by the authors.

The importance of assessing the risks of multimodal cargo transportation, especially with the use of maritime transport, has long been raised in the scientific literature. Moreover, various approaches have been used, from fairly original ones, using the “ecosystem approach” [3] to more classical ones, which capture the importance of the problem, but do not offer reliable mathematical approaches to solve it [4,5,6]. There are original developments using genetic algorithms, for example, as in the work of Xiong G. [7] This study used a multilevel genetic algorithm for routing with time windows and two objective functions, in particular, with delivery time optimization. The problem of low relevance of the solution by this method is to find the Pareto optimum and, most importantly, the need to use different mathematical tools to simultaneously calculate deterministic, stochastic and fuzzy parameters characteristic of cargo transportation are not taken into account. 

In fact, most of the studies presented in the scientific literature do not take into account the peculiarities of multimodal cargo transportation. These studies are often formed for special, separate options for improving multimodal transportation, for the search of intermediate, auxiliary options of objective functions. Thus, Fang X. [8] used a synergistic approach to find the best value of transportation in terms of meeting several economic components: innovation, management, business operations and information components, using an indicative approach and factor analysis. Harris’s work [9] is devoted to the improvement of multimodal transportation by optimizing only one component, which is emphasized by Fang X. [8], namely, information and communication technologies. The proposal to optimize multimodal transportation, according to Harris [9] is the formation of a single managed information space and the creation of a single integrated system for both transportation of goods and traffic management at all stages of transportation routes. Agamez-Arias [10] expands Harris’s thesis [9] on the importance of improving the information and communication system by forming links between system operators for the best logistics of intermodal transport systems. Boschian [11] studied the use of the metamodeling variant; for this purpose, a reference model and a library of simulation model variants are being developed. The unpredictability of risky situations is problematic in the use of this option in practice, with a sharp increase in the degree of risk and the need for significant time resources to optimize transportation routes. Verduzco-Garza [12] analyzed the options for finding the best route and a hybrid model of nonlinear mixed programming was selected as a basic one. To implement this model, the Lagrange method and evolutionary algorithms were used. Unfortunately, Verduzco-Garza [12] did not consider the use of simultaneously deterministic, stochastic and fuzzy parameters that determine the objective function. Quddoos [13] proposed a method of finding a compromise variant of the route by determining the weight of different possible variants. In [14], multi-objective linear programming is used to minimize the distances between ideal objectives to a possible target space by transforming intermediate targets into constraints. Kamal [14] also uses a balanced method of programming goals and minimizing the distances between ideal goals to a possible target space by solving subtasks of the general problem of linear programming. In Fleischmann’s article [15], various variants of mathematical modeling and a search for optimal solutions of reverse logistics are considered. Lam Shao-Wei [16] uses one of the most effective methods mentioned in [15], an approximate approach of dynamic programming, to quickly solve the problems of reverse logistics to move empty containers for multimodal transportation of goods. The paper uses a dynamic stochastic model of two port systems of two voyages of cargo containers. Wolfinger [17] used an iterative local lookup to optimize multimodal extended routes as part of column generation to optimize the objective function of the transportation cost. Möller [18] thoroughly analyzed various mathematical approaches to find the most efficient solutions for cargo transportation. Corman F. [19] proposed a dynamic balanced model. The influence and sensitivity of different parameters of the model on making strategic decisions regarding the objective function of freight traffic have been studied. Mokhtari [20] pointed out the growing importance of risk assessment for strategies based on this assessment and proposed a risk management methodology as a holistic system. The importance of transport risks and their impact on the main objective functions of cargo transportation, and competitiveness in particular, was also studied by Bubnova [21]. The approaches to the solution of the problem [1,2,3,4,5,6,7,8,9,10,11,12,13,14,15,16,17,18,19,20,21] analyzed in detail do not correspond to such peculiarities of the problem of multimodal transportations as efficiency of calculations and their multi parametricity. The main remark is that each of the considered methods a priori uses only one type of parameter of the objective function, either deterministic or stochastic. That is, it does not meet the condition of multi parametricity characteristics of multimodal transportation.

Modern mathematical methods for finding optimal solutions using multiparametric objective functions (Salas-Molina [22], Ballestero [23], Carosi [24]) also do not consider the use of deterministic, stochastic and fuzzy parameters simultaneously. For some types of parameters—for example, fuzzy ones—there are detailed studies, in particular, by Jasiulewicz-Kaczmarek [25], Rostamzadeh [26]. No such studies were found for all these groups.

The rest of the paper is organized as follows. In Section 2, the problem of transportation optimization for deterministic, stochastic and uncertain risks is discussed. In Section 3 the mathematical formulation of the problem is considered. The main work focuses on the creation of the optimization algorithm. In Section 4, an example of the practical use of the proposed mathematical model and results are presented. In Section 5 the results are discussed and the conclusion is drawn.

## 2. Statement of the Problem of Optimization of Multimodal Transportation for Deterministic, Stochastic and Uncertain Risks

The main remark is that each of the considered methods a priori uses only one type of parameter of the objective function—either deterministic or stochastic. That is, it does not meet the condition of multi parametricity characteristics of multimodal transportation.

Thus, despite the large amount of research, the task of finding the best alternative route for transport remains relevant when the risks on the previously chosen route increase certain limits and if the parameters that determine the objective function can be deterministic, stochastic and fuzzy quantities at the same time. This is especially important for regions where risks can take unacceptable values for a relatively short period of time.

Obviously, if the risks of transportation by a certain route or its stage increase, the task of a possible change of the further route arises and this requires the choice of an alternative route that meets the predetermined conditions. It is necessary to choose the best from a set of options according to a certain algorithm. Typically, such options are made in advance and the path is optimized using computer technology of the required power and with an unlimited resource of time. However, there may be a significant increase in the risks of transportation unexpectedly and within a relatively short time. The multimodal transportation of goods using maritime transport in the Black and Azov Seas may serve a relevant example of such a situation. When assessing the risks of maritime transport in the Black and Azov Seas through the seaports of Ukraine, a situation may arise where the risks of transportation may increase significantly within a relatively short time. One of the biggest threats is the military conflict between Ukraine and Russia [27,28,29]. However, there may be other short-term reasons, such as the restriction of cargo handling of a seaport by the railway monopoly of PJSC “Ukrainian Railways”, which has happened many times. There are other risks too. Moreover, the threat may increase when the cargo to one or another of the ports of Ukraine may already be on the way. Then, there is the problem of rapid reorientation of cargo to other ports, and, accordingly, to other routes, other modes of transport connected to those ports, and so on.

In previous scientific works [27,28,29], the risks of transportation of goods by sea were analyzed in detail and classified, so we will not return to these issues in this work. Moreover, in the mentioned works [27,28,29], mathematical approaches to the search for alternative variants of routes due to the growth of risks were offered. Experience with the practical application of these mathematical methods has shown that they require a significant amount of time and computer resources, so they cannot always be used in real time.

The problem of optimization of multimodal transportation is mainly reduced to the problem of finding the best value of the objective function from the studied set of options. The objective function is often understood as the main feature of the provision of freight services, the most important either from the point of view of its provider or from the point of view of its consumer. Such a feature may be the minimization of the prime cost (or cost) of transportation; transportation time; reliability of transportation and storage of cargo; minimization of threats and risks of transportation, etc.

We propose an auxiliary objective function for the mathematical implementation of the optimization algorithm: information entropy, in the sense of Shannon. The condition for its application is that the increase in the level of risks from the deterministic basic variant forms the level of information uncertainty, i.e., the growth of information entropy. As the study showed, the increase in entropy correlates with other objective functions, in particular, with increasing transportation time due to lengthening routes, additional loss of time for transshipment, increased transportation costs, reduced transportation reliability and cargo preservation.

Thus, the peculiarity of the problem in these circumstances lies in two main conditions. First, the calculations must be carried out quickly, because too-long calculations mean a delay for the vehicle on the road, which is not cheap and is not always safe. Secondly, the search for the best solution for choosing the best alternative route depends on various parameters: deterministic, stochastic, fuzzy quantities, the measurement of scales which are incompatible.

That is, the calculation of the functions of these groups of parameters requires the use of different mathematical tools. The use of various mathematical tools significantly complicates the problem both in terms of its formulation and the solution algorithm, and in terms of automatically greater use of computer resources, in particular, time for calculations. This requires finding new mathematical methods for finding the best alternative routes for cargo transportation.

Therefore, the task was to propose an algorithm that would satisfy these conditions. Mathematical methods of set theory, fuzzy logic and fuzzy relations, probability theory, context-dependent binary relations and graph theory are used in the work. For a practical application, statistical data of the State Statistics Service of Ukraine [30] and the Association of Seaports of Ukraine [31] were used as a basis for calculations.

## 3. Mathematical Formulation of the Problem

After analyzing the most commonly used objective functions for optimizing multimodal transportation of goods, in particular minimizing the prime cost (cost) of transportation, transportation time; reliability of transportation and storage of cargo, minimization of threats and risks of transportation, etc., it is revealed that the groups of parameters that determine them are overlapping sets. That is, some of the parameters can define several objective functions at the same time and others can define only one of them. The presence of such sets is characteristic of multicriteria problems, and the peculiarities of working with them are discussed in detail in [22,23,24]. Therefore, before selecting the objective functions that will be further developed, the whole set of parameters that characterize (or can be described in the future) multimodal transportation of goods in a particular region is determined. This set is defined as the space of variables Q, and the spaces of variables that define individual objective functions, and are part of the total space of variables are denoted, respectively, as $q_{1},\;q_{2},\;q_{3}\ldots q_{n}$. Then:

(1) $$q_{1},\;q_{2},\;q_{3}\ldots q_{n}⫋Q$$

That is, $q_{1},\;q_{2},\;q_{3}\ldots q_{n}$ are eigenvalues of the set Q. In the general case, these sets meet the condition:

(2) $$q_{1}\cap q_{2}\cap q_{3}\ldots \cap q_{n}$$

Let’s analyze the problem statement using the apparatus of set theory. In this case, Q is, by definition, a superset, and $q_{1},\;q_{2},\;q_{3}\ldots q_{n}$ are subsets. Since none of the subsets contain empty elements, neither does the superset. However, each of the sets of risks $\varepsilon_{i}$ for each *i*-th element of the multimodal transport route may contain an empty set. At the same time, each of the risk sets for each route element is not an empty set:

(3) $$\left\{\varepsilon_{i}\right\}\notin \emptyset$$

For a superset, the space of variables Q is also true as follows:

(4) $$Q-\overset{n}{\underset{1}{\sum }}q_{i}=\emptyset$$

Involvement of the set theory apparatus to operate on the space of variables allows us to algorithmize the exclusion of possible errors in the subsequent stages of optimization. For example, in the general formulation of the problem of optimization of multimodal transportation, it is impossible to exclude the optimization of several objective functions, for example, the option of minimizing the cost and time of transportation simultaneously.

Therefore, the use of set theory at the stage of the algorithm to determine the required set of objective functions for their subsequent optimization, forms a limitation of the problem, in particular excludes actions on operations with variable space, which is subject to certain restrictions and which, accordingly, can lead to errors in optimizing these functions. In particular, when optimizing one of the objective functions, it should be borne in mind that its variable parameters may be included in a subset, which determines the other objective function.

In each subspace of variables $q_{i}$ we distinguish the deterministic $q_{i}^{d}$, the stochastic $q_{i}^{s}$ and the fuzzy $q_{i}^{f}$ part of the variables. We find the corresponding response functions in these parts $f_{i}^{d}$, $f_{i}^{s}$, $f_{i}^{f}$ [32].

Next, we use orthogonal tensor analysis for the transport network of the country, which is an improved extension of the approach proposed in the works [27,28,29].

We consider the system to be orthogonal, because it can be considered as a set of routes, which, by definition, can be topologically closed or open, which is, in fact, a sign of orthogonality.

Next, the algorithm has two alternatives. One can reduce the analysis to contour or nodal methods. This will depend on where the traffic jam is to be envisaged—in the network nodes that are considered as seaports, or in the contours—as railroad, road, river paths connected to transit nodes. In accordance with the chosen method, the transformation tensor (or transition matrix) must be determined, then the threshold intensities of cargo arrival, the intensity of passage through nodes or contours and the speed (intensity) of its handling must be set.

However, as the analysis shows, the peculiarity of the transport network of Ukraine is such that the most vulnerable to cargo delays are the joints of the node and circuits. This is due to transhipment operations.

Therefore, an alternative approach to the choice of nodal or contour methods is proposed. To simplify the implementation of the algorithm, we proposed to consider the transport network as a set of linearly independent contours and double nodes (so-called “sections”).

The use of tensor analysis is convenient for the simultaneous use of stochastic, fuzzy and deterministic variables, in particular, given the invariance of tensors. Representation of deterministic variables using tensor analysis by a matrix that can be considered as a projection of the tensor is obvious. Representation of stochastic variables is described in [2].

Higher transport risks increase the uncertainty of the objective function, which can be seen as an increase in entropy in Shannon’s representation. For stochastic parameters $\vec{x}$, the corresponding value of this entropy is the mathematical expectation $E\left[\vec{x}\right]$. As it is known, the mathematical expectation of a stochastic vector quantity is equal to a vector, the components of which are the mathematical expectations of the components of the specified stochastic vector:

(5) $$E\left[\vec{x}\right]={\left(E\left[x_{1}\right]\ldots E\left[x_{n}\right]\right)}^{T}для\;\vec{x}={\left(x_{1},\ldots \;x_{n}\right)}^{T}:U\to R,\;f_{i}^{s}=f_{i}^{s}\left(\vec{x}\right)$$

For fuzzy variables, the algorithm is as follows. We define a fuzzy set as intuitionistic (IFS), which is defined on the space U by fuzzy variables of the function $f_{i}^{f}$. It should be pointed out that IFS on U is represented as a set of ordered triplets. The components of these triplets are: the element of space, the membership degree M, the non-membership degree N so that M+N≤1; M,N∈[0, 1]. For the limit value of the sum M+N=1 we obtain a fuzzy set. For cases M+N<1 we obtain uncertainty.

This uncertainty is interpreted by us as information entropy $S_{IFS\;}=1-\left(M+N\right)$.

In this case, the main condition for setting the problem with fuzzy variables is to minimize the information entropy: $S_{IFS\;}\to min,S_{IFS\;}\in opt\left(f_{i}^{f}\right)$.

The use of tensor analysis for fuzzy variables makes it possible to apply their representation by the expression 3×n де n=1, 2, 3…, which leads to the possibility of using the principle of the entropy extremum to unambiguously determine the membership function. Since the methods of tensor analysis make it possible to decompose the tensor function into a group of attached tensors, it allows us to obtain more complete information about fuzzy variables.

To represent fuzzy variables with a membership function in the form of Gaussian triangle or trapezoid, we use dyad tensors. We propose to represent a fuzzy tensor variable by Kronecker pairwise multiplication of the corresponding variable by the membership function. When there are two one-dimensional vectors $\vec{x}=\left(x_{1},x_{2}\ldots x_{n}\right)$. and a ${\vec{\mu }}_{x}=\mu_{x}^{1},\;\mu_{x}^{2}$ … $\mu_{x}^{2}$ fuzzy tensor variable is found as $T_{x}=x\mu_{x}^{T}$, $\mu_{x}\to \left[0,\;1\right]$, where T is the transposition symbol.

That is, this approach allows us to use of tensor analysis for all possible types of variables, formally perform all algorithms with them, apply the analysis of invariants instead of fuzzy tensor representation, and, most importantly, use the property that follows $tr\;\left[x_{i}\right][\mu_{i}^{x_{i}}$] and coincides with the defadified value $\vec{x}$ ~ $\left(\vec{x}\right)=\sum_{i=1}^{n}x_{i}\mu_{i}^{x_{i}}\sum_{i=1}^{n}\mu_{i}^{x_{i}}$.

Next, the fuzzy tensor variable is used when performing arithmetic operations according to the algorithm. To do this, we consider it as a matrix *n* × *n*. As it is known, arithmetic operations on fuzzy quantities have matrix analogues. In accordance with the principle of fuzzy extension, we use standard methods and models for fuzzy representations.

With the increase of risks on the group of stages of transport routes (let’s call them the basic group, abbreviated, *bg*, which is determined by the matrix $Q_{bg}\in \nabla^{m\times n},\;where\;m=m_{1}m_{2},\;n=n_{1}n_{2}$) outside the set limits, there is a problem in finding a new group of stages with acceptable values of risks and their integration to the status of routes with relatively safe stages of the base group, i.e., the formation of matrices $Q_{1}\in \nabla^{m_{1}\times n_{1}}$ and $Q_{2}\in \nabla^{m_{2}\times n_{2}}$, close to $Q_{bg}$ according to the algorithm.

Thus, the problem is reduced to:

(6) $$\emptyset \left(Q_{1},\;Q_{2}\right)=\|Q_{bg}-Q_{1}Q_{2}{\|}_{F}=\|{\hat{Q}}_{bg}-vec\;(Q_{1})vec{\left(Q_{2}\right)}^{T}{\|}_{F}=min$$

where ${\hat{Q}}_{bg}$ is a vectorized matrix of the base group, and the vectors ($vec{\left(Q_{1}\right)}^{T},vec{\left(Q_{2}\right)}^{T}$) form a subset of ordered pairs.

In this case, Equation (6) can be solved as bilinear, by the method of least squares. However, this will complicate and prolong the calculations. In the case when we fix $Q_{1}$ or $Q_{2}$, the problem is linearized and with respect to $Q_{2}$ or $Q_{1}$ we obtain the solution:

(7) $$\{vec\;(Q_{1}^{\left(opt\right)})={\left(\sigma_{1}\right)}^{1/2}\;U\left(:1\right),\;f_{i}^{f}\to opt\;vec\;(Q_{2}^{\left(opt\right)})={\left(\sigma_{1}\right)}^{1/2}\;V\left(:1\right),f_{i}^{f}\to opt\;$$

A set of risk factors was analyzed in a previous article on this issue [28]. The set of risk factors [28] includes the following risks by groups:

- Natural risks (weather or biological factors that cause damage to cargo; natural disasters);
- Technological risks (damage to cargo caused by overloading and transportation; depletion of equipment; computer and information dangers; fires, accidents);
- Political risks (military and political instability; economic sanctions, closure of borders);
- Legal and Tariff Risks (amendments to laws and legislation that regulate the carriage of goods; changes in tariffs);
- Commercial risks (customers refusing to collect or pay for the transportation of the goods; changes in the cost of transportation after the contract is signed; non-fulfillment of contract terms);
- Financial risks (change in the exchange rate; lending threats; inflation threats);
- Social risks (intentional damage or destruction of the goods: territorial conflicts; air strikes or potential threats of it occurring).

Risk assessment according to the algorithms proposed in [27,28,29,33] allows us to identify the stages of routes where the risks are excessive in the first stage. The degree of risk for one or another objective function is considered to be excessive when the factor it optimizes—cost (С), time (τ) of transportation or another one—becomes bigger than the threshold value ($С\geq {С}_{max},\;\tau \geq \tau_{max}$). These routes and their stages are further excluded from consideration. As a rule, the stages with the so-called “absorbing risks” are super large. According to [29,34], “absorbing risks” are the risks of escalation of hostilities, closure of waters for transport vessels, etc., i.e., risks that weigh more than the total value of all other types of risk [29]. The next step of the algorithm is to identify those stages of routes where the weight of risks that have the greatest impact on the objective function is greater than others. These routes and their stages are also further excluded from consideration, which significantly reduces the volume of calculations. Thus, the construction of the mentioned graph G is carried out in accordance with the method described in [27,35].

The best compromise is to find a direct or indirect connection between the chosen objective functions. For example, some routes may be worse in terms of prime cost or time of transportation. However, in the case of a probable increase in the level of risks at the stages of these routes during transportation, they may be the best. To expedite the assessment of such options, the scale of change of each type of risk should have some functional comparison with the prime cost, time of transportation or other objective functions.

The last step of the algorithm is the linearization of data in accordance with the Ehrgott proposition [1,2]. Indeed, for a significant interval of change of the objective function, the route system can be affected by different risks, so reducing the intervals or splitting a large interval into a number of small ones will reduce the effects of other risks and separate the impact of one risk or a small group of them.

## 4. Results

**Figure 1:**
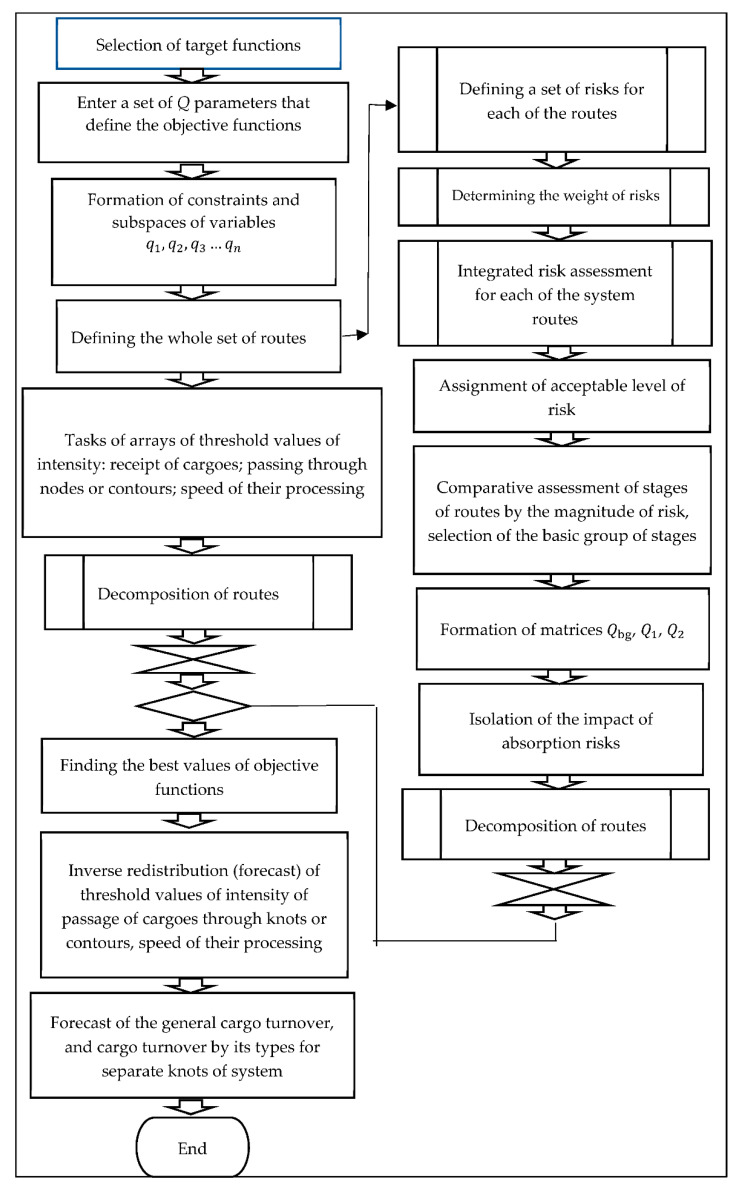
Block diagram for the basic structure of the entropy method.

For an example of practical use of the proposed mathematical model (a block diagram of the mathematical model is shown in Figure 1), we do not give its application in real time for the transportation of commercial goods, as it is associated with both trade secrets and is a narrow utilitarian application, which is uninteresting to the general public, but analyze and forecast redistribution of load for a longer time interval for transport hubs—seaports of Ukraine as transit points of multimodal transportations. To do this, we use the results of calculations given in [27,36] as reference values of risks. As reference data for calculations, we also use the above-mentioned sources of official statistics: the State Statistics Service of Ukraine [30] and the Association of Seaports of Ukraine [31]. In the block diagram shown in Figure 1, program blocks for the use of algorithms [27,28] are marked as “standard”. The original blocks offered in the presented article are marked by single rectangles, that is, as non-standard programs. This allows us to illustrate the part of the use of the general methodology in the block diagram with the marking of the part, which is presented in the article.

**Figure 2:**
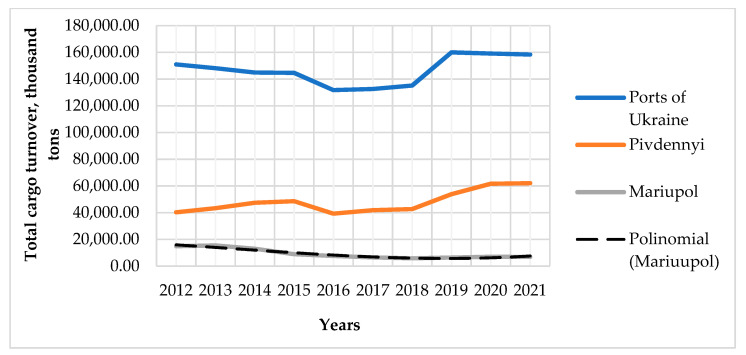
Total cargo turnover for all seaports of the country, the ports of Mariupol and Pivdennyi.

We will analyze and forecast for the port of Mariupol on the Sea of Azov, the largest port in the east of the country, for which the so-called “absorbing” risks [27,37] of military conflict are of the greatest importance; the port of Pivdennyi, for which these risks correspond to the level of risk in the country as a whole; and the port of Izmail, one of the ports in the west of the country, for which the risks of military conflict have the lowest level and coincide in weight with other risks, which we will call the background—standard risks of cargo transportation. The total turnover is shown in Figure 2. The analysis allows us to establish that the ratio of cargo transportation for the port of Mariupol from 2012 to 2018 decreased 2.53 times. The beginning of the active phase of hostilities falls on 2014. The volume of traffic for the port of Mariupol in 2015 decreased by 31%.

The increase in the level of risks for the port of Mariupol led to a redistribution of cargo turnover of this port to other ports of the country, for which the risks of military action, given the coincidence of the usual, “background” risks, were lower. Thus, for the port of Pivdennyi it is clear from Figure 2 that with a small lag in time, after the fall in traffic in 2015, when the risk of military conflict for all ports of Ukraine was the same, the port of Pivdennyi not only restored the transportation load to the level before hostilities, but also increased it 1.57 times during the period 2016–2020, when the level of threat of military actions for the port decreased almost 10 times.

**Figure 3:**
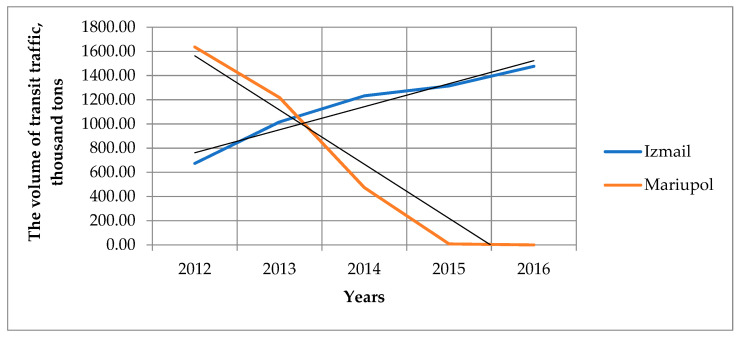
Transit cargo transportation for the ports of Mariupol and Izmail for the period 2012–2016.

Presented in Figure 1, the “Block diagram for the basic structure of the entropy method” illustrates the implementation of the mathematical model described in detail in Section 3. As it can be seen from Figure 3, the program blocks of the entropy method are connected with program blocks of the tensor method [27,28,29]. The coordination of the blocks takes place at the level of data matching after processing the results in the blocks “Route decomposition”. The result of the algorithm is the inverse redistribution (forecast) of the threshold values of the intensity of the passage of goods through nodes or circuits, their processing speed and, if necessary, the forecast of total turnover and turnover by its types for the system and its nodes, such as ports.

The analysis shows that part of the cargo, the transportation of which was previously provided by the port of Mariupol, passed through the port of Pivdennyi. That is, the route of multimodal transportation for certain types of cargo through the port of Pivdennyi has become an alternative to the route through the port of Mariupol, due to the much greater risks of transportation through the port of Mariupol. This has led to a certain increase in the cost and time of transportation of goods from Mariupol and adjacent areas to the port of Pivdennyi. However, as the analysis showed, this growth did not exceed acceptable limits. According to the proposed method, we linearize the transportation load, which is why we will consider it for shorter periods of time. The linear approximation is performed according to equations in the form: *y* = *ax* + *b*. The coefficient (*a*) is known to be the slope ratio of the approximation line in the corresponding coordinates. Correlation analysis applied to risk levels and coefficients (*a*) of linear approximations of transportation load through the port of Mariupol for the respective time intervals and the average value of integrated traffic risk for this period indicated a significant level of their correlation (Table 1).

The comparison of the forecast results with the actual data of the total cargo turnover of the ports of Mariupol and Pivdennyi and transit cargo turnover of the port of Izmail is given in Table 2. The comparison indicated an expected increase in the relative error of predictions using the entropy method, which was used to obtain results in the shortest possible time, less than the time of using the tensor model [27]. Such an increase in error in most cases is satisfactory for the prompt change of routes, estimation of time spent on transportation of goods and the cost of transportation.

This has greatly simplified the analysis and forecasting of multimodal transportation.

If we analyze transit transportation, which is part of the general cargo turnover of seaports, it is a more typical example of multimodal transportation due to its nature. Analysis of transit transportation for the ports of Mariupol and Izmail for the period 2012–2016 indicates the opposite trend in their dynamics—the coefficients of approximation of linear trends for them are opposite in sign, respectively 190.37 for the port of Mariupol, 448.04 for the port of Izmail (see Figure 3).

The volume of transit traffic for the port of Mariupol from 2015 to the present is zero. That is, the port of Izmail has become an alternative option for the transportation of transit cargo after the risk for the port of Mariupol reached an unacceptable value for the integrated objective functions: the prime cost of transportation, transportation time and cargo safety.

The general algorithm for forecasting transit traffic was the same as for the total cargo turnover of the ports of Mariupol and Pivdennyi (see Figure 1) as transit traffic is a part of total cargo turnover. And as a result, data on a particular type of transportation will be obtained on the condition of the introduction of appropriate restrictions on the choice of data when setting arrays of threshold values of the cargo intensity, passing through nodes or circuits, the speed of its processing, etc.

According to the 2020 data, the relative error of the forecast for the port of Izmail is higher than the forecast for other ports: 5.645% for the entropy method. However, as the analysis for Izmail shows, it is higher than that of other ports and the forecast by the tensor method is −3.732%. The explanation for this is that the volume of transit cargo turnover of the port of Izmail is much less than the total cargo turnover of the ports of Mariupol and Pivdennyi. Therefore, the total impact of ordinary risks for Izmail in absolute terms reduces the accuracy of the forecast.

**Figure 4:**
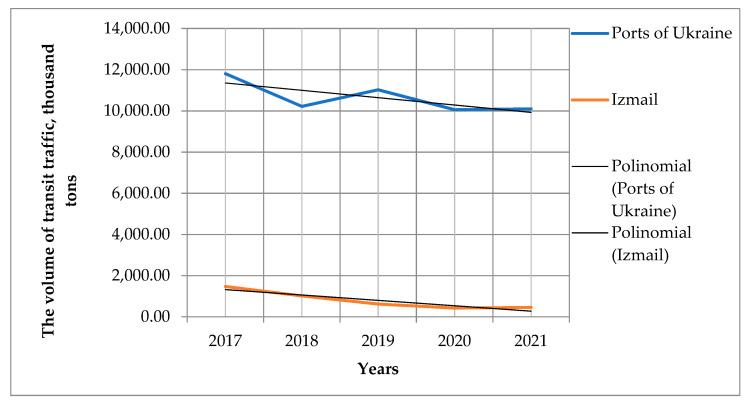
Transit cargo transportation for the ports of Mariupol and Izmail for the period 2017–2021.

A comparison of transit cargo volumes for the period 2017–2020 for all ports of Ukraine and the port of Izmail shows that the slope ratios of linear trends for them not only coincide in sign, but also almost coincide in value (Figure 4). It is 444.12 for all ports of Ukraine, 453.73 for the port of Izmail. This indicates that for this period the level of background transport risks for all ports of Ukraine and for the port of Izmail in particular are very close in value.

**Figure 5:**
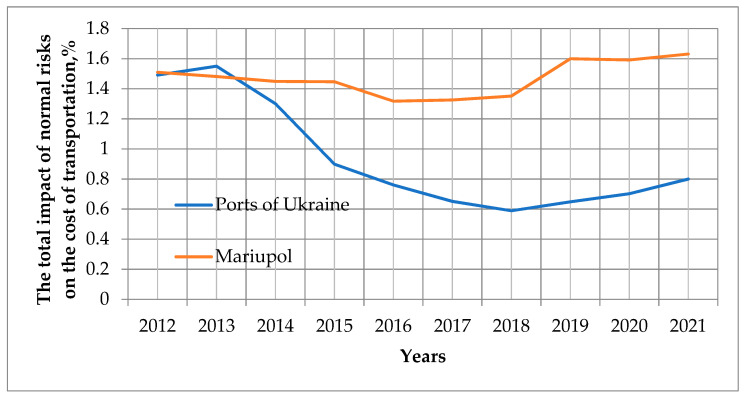
The total impact of normal risks on the cost of transportation for all ports and the port of Mariupol for the period 2012–2020, %.

The approach illustrated in Figure 5 is an example of estimating the total impact of normal risks on the objective function. The result of the analysis proves that the main risk for changing routes was the risk of hostilities. All other risks, as it is shown by the comparison of the total impact of normal, background risks on transportation costs for all ports of Ukraine and the port of Mariupol for the period 2012–2020, are commensurate (see Figure 5) or short-term, and, as it is shown in the figure, these risks do not correlate with each other. Risk calculations were performed according to the method presented in scientific papers [27,28,29]. This also illustrates the use of the express method of rapid selection of important risks for assessment.

Thus, the general analysis of the volume of traffic through the ports of Ukraine indicates the presence of a factor that forces a change in the traditional routes of transportation. This factor is a significant risk, the magnitude of which is much greater than the impact of all other risks. These risks in [29] are called absorbing, because their impact absorbs the combined impact of other risks. As the analysis showed, for relatively long time intervals (one to three years), the constant value of the absorbing risk for the seaports of Ukraine is the risk of military action. All other absorbing risks are short-term (a week or less).

The presence of the risk of hostilities and the variability of its magnitude for different ports of Ukraine leads to a redistribution of transportation routes among the ports. These ports are selected by shipping carriers in accordance with the intended functions by which they are guided. The port of Pivdennyi mainly provides transportation of general cargo, which was previously transported through Ukrainian ports on the Sea of Azov, because this transportation is slightly higher in terms of prime cost and time of cargo transportation than the corresponding indicators for the Azov sea ports. Some of the transit multimodal cargoes, the transportation of which was stopped through the ports of the Sea of Azov, began to pass through the port of Izmail. Thus, it can be indicated that the proposed mathematical model is useful not only for real-time traffic management, but also for estimating and forecasting integrated traffic volumes through Ukrainian seaports.

## 5. Conclusions

This paper proposes a mathematical model of the optimal choice of alternative routes or stages of multimodal transportation routes in case of increased risk on existing routes or subsequent stages of transportation on the condition of certain threshold values. The peculiarities of the proposed mathematical model are its efficiency and the ability to process risks, the parameters of which can be deterministic, stochastic and fuzzy quantities simultaneously. Furthermore, this model allows finding a compromise solution using several objective functions and in cases where the sets of parameters that define these functions overlap. The efficiency of choosing the best alternative according to the proposed algorithm allows us to solve the problem, even if it occurs during the start of transportation, i.e., in real time. This is especially important for the so-called absorbing risks, the magnitude of which may suddenly increase to an unacceptable value and these risks, by weight, will be much greater than all other present risks.

Choosing not only a possible alternative path, but also the best possible, is very important in such force majeure situations. Such an absorbing risk, which is not short-term (for a period of less than a week) but long-term (for years) is the risk of military action for Ukrainian realities. This risk is different for different hubs of multimodal transport routes, which traditionally include seaports. Therefore, the presence of this risk leads to a redistribution of traffic among ports.

The analysis showed that in these circumstances all other risks are background, their total impact on the objective function of transportation is insignificant or short-term.

As it is shown, this mathematical model can be used for both short-term and longer-term forecasting.

This allowed forecasting the volume of transportation, the magnitude of background risks, the volume of transit traffic for ports with a significant discrepancy in the value of absorbing risk for the coming year. The practical approbation of the developed mathematical model indicated its possible use both for practical purposes and for theoretical research of multimodal transportation, congestion of transport hubs and redistribution of transport flows in crisis conditions.

This study is a continuation of the scientific work, the results of which were published earlier [27,28,29]. The improvement was carried out according to the directions predicted by the authors and in response to the appropriate comments received during the discussion, which continued after the first publications on this topic. The practitioners’ remark was that when transporting cargo that is already on a multimodal route, there is always a lack of time and computer resources to conduct detailed optimization studies of alternative routes and to choose the best one. It is indicated that after identifying the risk and identifying a set of risks specific to each of the routes, the proposed mathematical apparatus should not be used fully, according to the algorithm, and, if possible, standard procedures and options can be used to assess an alternative route according to the objective function or a limited group of them. These options can form a library that can be refined dynamically. The algorithm for dynamic updating of such a library and its structure are currently under development. This can really speed up the optimization procedure in some way. Research in this direction will be continued.

## Figures and Tables

**Table 1:** The degree of correlation of the coefficients of linear approximation of the total cargo turnover through the port of Mariupol for the time interval and levels of integrated risk.

Parameter		The Absolute Value of the Coefficient in the Approximation Equation	The Magnitude of the Integrated Risk
Years	2013–2015	3257.7	0.63
	2016–2019	1038.0	0.27
	2019–2020	499.66	0.149
Correlation coefficient		-	0.998257

**Table 2:** Relative error of the forecast of the basic model and entropy method according to the data of 2020.

Port	Forecast Using the Base Model, Thousand Tons	Forecast Using the Entropy Method, Thousand Tons	Actual Results, Thousand Tons	Relative Error of the Base Model, %	The Relative Error of the Entropy Method, %
Mariupol	6988.7	6952.2	7025.43	−0.523	−1.042
Izmail	412.5	404.3	428.49	−3.732	−5.645
Pivdennyi	47,512.2	47,145.4	47,629.48	−0.246	−1.016

## Data Availability

Data sharing not applicable.
